# Impact of tea leaves categories on physicochemical, antioxidant, and sensorial profiles of tea wine

**DOI:** 10.3389/fnut.2023.1110803

**Published:** 2023-02-07

**Authors:** Chun Zou, De-Quan Chen, Hua-Feng He, Yi-Bin Huang, Zhi-Hui Feng, Jian-Xin Chen, Fang Wang, Yong-Quan Xu, Jun-Feng Yin

**Affiliations:** ^1^Tea Research Institute Chinese Academy of Agricultural Sciences, National Engineering Research Center for Tea Processing, Key Laboratory of Tea Biology and Resources Utilization, Ministry of Agriculture, Hangzhou, China; ^2^Graduate School of Chinese Academy of Agricultural Sciences, Beijing, China; ^3^School of Pharmacy, Jining Medical University, Jining, China; ^4^College of Tea Science, Guizhou University, Guiyang, China

**Keywords:** tea wine, organic acids, volatile components, catechins, antioxidant activity, sensory properties

## Abstract

**Introduction:**

Tea is the main raw material for preparing tea wine.

**Methods:**

In this research, four types of tea wine were prepared using different categories of tea leaves, including green tea, oolong tea, black tea, and dark tea, and the comparative study looking their physicochemical, sensorial, and antioxidant profiles were carried out.

**Results:**

The dynamic changes of total soluble solids, amino acids and ethanol concentrations, and pH were similar in four tea wines. The green tea wine (GTW) showed the highest consumption of total soluble solids and amino acids, and produced the highest concentrations of alcohol, malic, succinic, and lactic acid among all tea wines. The analysis of volatile components indicated the number and concentration of esters and alcohols increased significantly after fermentation of tea wines. GTW presented the highest volatile concentration, while oolong tea wine (OTW) showed the highest number of volatile compounds. GTW had the highest total catechins concentration of 404 mg/L and the highest ABTS value (1.63 mmol TEAC/mL), while OTW showed the highest DPPH value (1.00 mmol TEAC/mL). Moreover, OTW showed the highest score of sensory properties.

**Discussion:**

Therefore, the types of tea leaves used in the tea wine production interfere in its bioactive composition, sensorial, and antioxidant properties.

## 1. Introduction

Tea wine is an alcoholic drink with tea as the main raw material (1). It combines the fragrance of tea and mellow of wine, which has a unique and attractive flavor (2, 3). Moreover, there are many potential benefits of tea wine for human health, including immunity improvement, neuroprotective effect, antioxidant effect, and antimicrobial efficacy (4–6). Therefore, tea wine has attracted a wide attention of researchers and consumers in recent years (7).

In tea wine fermentation, sugar is supplemented as carbon source, while tea is not only used as nitrogen source, but also provides flavor and functional components for tea wine (8, 9). According to different fermentation degrees (10), tea leaves have the categories of green tea (non-fermented), oolong tea (semi-fermented), black tea (full-fermented), and dark tea (post-oxidized with microorganisms). The major polyphenolic catechins are largely retained in green tea, while they are enzymatically oxidized or metabolized by microorganisms to catechin polymers in black tea or dark tea. The major polyphenolic catechins (11), including (−)-epigallocatechin (EGC), (−)-epicatechin (EC), (−)-epicatechin gallate (ECG), (−)-epigallocatechin gallate (EGCG), (+)-catechin (C), (−)-catechin gallate (CG), (−)-gallocatechin (GC), (−)-gallocatechin gallate (GCG), are known to possess antioxidant activity (3). Moreover, the sensory properties and aroma compounds in various categories of tea are also different (12). Therefore, the effects of tea categories on the properties of tea wine need to be clarified.

In this study, four types of tea wine, including black tea wine (BTW), green tea wine (GTW), oolong tea wine (OTW), and dark tea wine (DTW) were prepared using different categories of tea leaves. The aim of this research was to investigate the effects of categories of tea leaves on the physicochemical, antioxidant, and sensorial profile of tea wines.

## 2. Materials and methods

### 2.1. Reagents and materials

Black tea (Keemun), green tea (Longjing), oolong tea (*Tieguanyin*), and dark tea (Pu-erh) were purchased from Yifutang Co. (Hangzhou, China), Longguan Co. (Hangzhou, China), Mingjuhui Co. (Quanzhou, China), and Xinyihao Co. (Kunming, China), respectively. Lyophilized yeast powder was purchased from Angel Yeast Co., Ltd. (Yichang, China). Sucrose was purchased from Jingtang Co. (Beijing, China).

Standards of organic acids (gluconic, succinic, citric, and gallic acid) and catechins (EGC, EC, ECG, EGCG, C, CG, GC, and GCG) were purchased from Sigma-Aldrich Shanghai Trading Co., Ltd. (Shanghai, China). Methanol and acetonitrile of high performance liquid chromatograph (HPLC) grade were purchased from Merck Co. (Darmstadt, Germany). All the other chemicals were of analytical grade and purchased from Sinopharm Chemical Reagent Co., Ltd. (Shanghai, China).

### 2.2. Tea wine production

The sugared tea infusion was prepared as the following steps: 225 g sucrose was dissolved in 1.5 L water. The solution was sterilized at 121°C for 15 min, and then 9 g of tea leaves were added and extracted at 90°C for 20 min. After extraction, the tea leaves were removed and the sugared tea infusion was transferred into a sterilized glass jar, which was cooled down to room temperature before inoculation.

For yeast strain recovery, 0.75 g lyophilized yeast powder was added into a 5% sucrose solution, and maintained at 35°C for 20 min. Subsequently, the recovered yeast was inoculated into the sugared tea infusion, and then tea wine fermentation was carried out at 28°C for 20 days (13).

### 2.3. Determination of total soluble solids contents

The total soluble solids content (TSSC) of tea wine were determined using a refractometer (RX-007a, Atago Co., Ltd., Japan).

### 2.4. Determination of ethanol

The ethanol concentration was determined using a biosensor analyzer (Jinan Yanhe Biotechnology Co., Ltd., Jinan, China). The biosensor analyzer was calibrated with the alcohol standard solution before measurement. If the alcohol concentration of sample was higher than 0.4 g/L, the sample should be diluted. Twenty-five microliters sample was sucked accurately using a micro sampler, and injected into the biosensor analyzer for enzyme reaction. After reaction, the test result will be displayed on the screen.

### 2.5. Determination of amino acids

The concentration of amino acids in tea wine was determined using a spectrophotometer by the ninhydrin method (14) at 540 nm. Glutamic acid was used as the standard.

### 2.6. Determination of pH

The pH value of tea wine was determined using a pH meter (SG2, Mettler-Toledo Instruments Co., Ltd., Shanghai, China).

### 2.7. Determination of organic acids and catechins

Organic acids (malic, succinic, lactic, and gluconic acid) were analyzed using a HPLC equipped with an Agilent ZORBAX® SB-C18 column (4.6 × 150 mm, 5 μm) and a UV-DAD detector (15). The mobile phase was a mixture of methanol and 1 g/L phosphoric acid (3:97). The detection wavelength was set at 220 nm. The column temperature and flow rate were maintained at 28°C and 1 ml/min, respectively.

Catechins (EGC, EC, ECG, EGCG, C, CG, GC, and GCG) were analyzed using a HPLC equipped with a Waters Symmetry C18 column (4.6 × 250 mm, 5 μm) and a UV-DAD detector (16). The detection wavelength was set at 280 nm. The mobile phase A was formed with 2% acetic acid, and the mobile phase B was 100% acetonitrile. The following elution gradient program was adopted: 0–16 min, 6.5% B; 16–25 min, 15% B; and 25–30 min, 6.5% B. The flow rate and column temperature were maintained at 1 ml/min and 35°C, respectively.

### 2.8. Analysis of volatiles

The tea wine samples were pretreated by headspace solid-phase microextraction (HS-SPME) using a SPME stable flex fiber (50/30 μm, PDMS/DVB/CAR) for the headspace experiments (17). One hundred milliliter of sample and 100 μl of internal standard (10 μg/ml ethyl caprate) were mixed and placed in a 150-ml sealed glass vial. After equilibration and stabilization for at 50°C 5 min, the SPME fiber was used for the absorption of volatiles for 40 min. After absorption, the volatiles were desorbed in a gas chromatography–mass spectrometry (GC–MS) injector at 220°C for 5 min.

The volatiles were analyzed with an Agilent 6890N GC equipped with 5975B mass selective detector. A DB-5MS (60 m × 0.25 mm × 0.25 μm) capillary column was used for the separation. The GC inlet temperature was set at 220°C. The carrier gas was set at 1.5 ml/min of high purity helium (99.999%). The temperature was programmed as follows: 50°C for 2 min, raised to 80°C at 3°C/min, held at 80°C for 2 min, then raised to 180°C at 5°C/min, held for 1 min, and finally raised to 230°C at 10°C/min and held for 2 min. For MS analysis, the electronic energy of the EI mode and the temperature of the ion source were set at 70 eV and 230°C, respectively. The mass scan range was 50–500 *m*/*z*. The volatile compounds were identified based on the National Institute of Standards and Technology (NIST) database library.

### 2.9. Analysis of antioxidant activity

The 1,1-diphenyl-2-picrylhydrazyl (DPPH) and 2,2′-azinobis-3-ethylbenzthiazoline-6-sulfonic acid (ABTS) assays were used to analyze the antioxidant capacity of tea wines, using trolox as a standard.

The DPPH assay was carried out as follows: 3 ml of DPPH solution (200 μM) and 1.5 ml of sample were mixed, and then setted in a dark place at room temperature for 30 min. Subsequently, the decrease in absorbance at 515 nm was measured.

The ABTS assay was conducted as follows: 7 mM ABTS solution and 2.45 mM potassium persulfate solution were mixed and kept in the dark for 12–16 h. The mixture should be diluted to an absorbance of 0.70 ± 0.02 at 734 nm before use. One milliliter of sample and 4 ml of ABTS diluted solution were mixed and kept for 6 min in a dark at room temperature. The decrease in absorbance at 734 nm was measured.

### 2.10. Analysis of sensory properties

The sensory properties of tea wines were scored by a trained team of eight panelists (four men and four women, 23–50 years old) from the Tea Research Institute of the Chinese Academy of Agricultural Sciences. Based on their preference, the panelists gave the scores for taste, odor, appearance, and overall acceptability. A scoring range of 1–9 was used, which indicated (1) extreme disliking, (2) great disliking, (3) moderate disliking, (4) slight disliking, (5) neither liking nor disliking, (6) slight liking, (7) moderate liking, (8) great liking, and (9) extreme liking.

### 2.11. Statistical analysis

All results were presented as mean ± standard deviation (SD) of three replicates. The level of statistical significance among the means was analyzed by one-way ANOVA using SPSS (version 18.0, SPSS Inc., Chicago, IL, United States).

## 3. Results and discussion

### 3.1. The changes of total soluble solids, amino acids, and ethanol concentrations during fermentation

**Figure 1:**
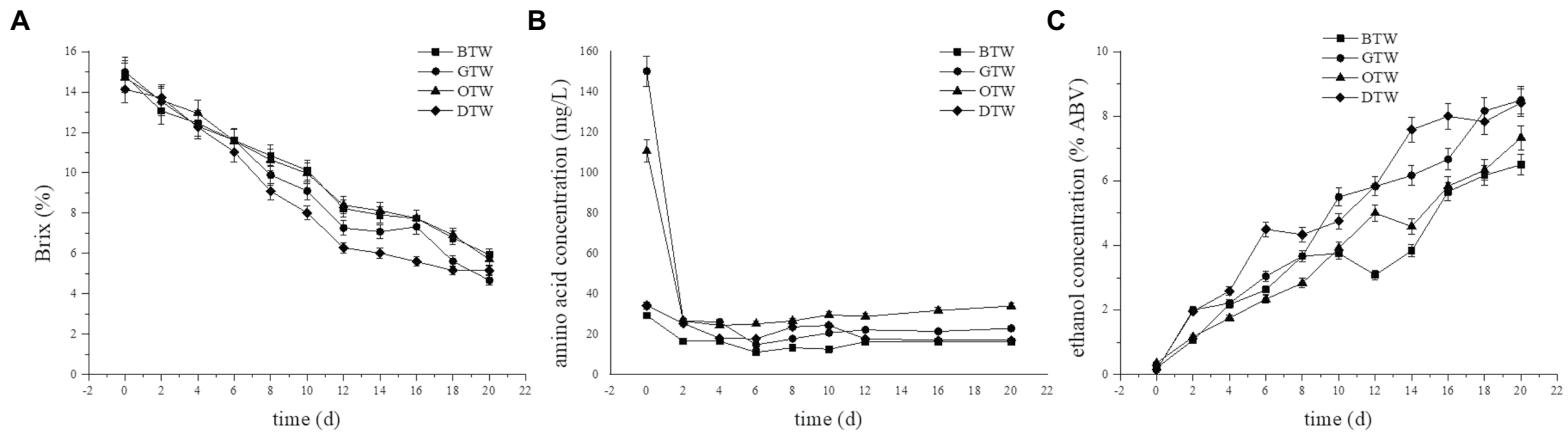
The changes of total soluble solids **(A)**, amino acids **(B)**, and ethanol **(C)** concentrations during the fermentation of tea wines.

The total soluble solids contents (TSSC) of all four tea wines were decreased with fermentation time (Figure 1A), and the TSSC of GTW showed the largest decline among all samples. The TSSC of GTW at 20 days reached 4.67°Bx, which was 22.3, 19.1, and 9.6% lower than that of BTW, OTW, and DTW, respectively. Similar range of TSSC at the end of wine fermentation was reported by Lu et al. (18) and Joshi et al. (8). Because sugar was the main contributor of the TSSC in tea wine (18), green tea might be more conducive to sugar consumption than the other three kinds of tea leaves.

Since there was no supplemental nitrogen source added in tea wine fermentation, amino acids in tea broth might be the main nitrogen source for cell growth and metabolism. As shown in Figure 1B, the amino acid concentrations of all four types of tea wine rapidly decreased during the first 2 days, and remained at a low level (<34 mg/L) after that. The initial amino acid concentration of GTW was 150 mg/L, which was the highest among all samples. Therefore, high amino acid concentration in tea broth might promote sugar consumption in tea wine fermentation. Similar results were found in the fermentation of soy whey alcohol (19) and wine (20).

Ethanol is an important metabolite of tea wine. As shown in Figure 1C, the ethanol concentrations of all four tea wines were increased with fermentation time. The ethanol concentration of GTW at 20 days was 8.5%ABV, which was 1.31-fold, 1.16-fold, and 1.01-fold than that of BTW, OTW, and DTW, respectively. This indicated that the alcohol yield of GTW was significantly higher than that of BTW under the similar initial sugar concentration, and similar results could be found in previous studies (21, 22).

The dynamic changes of total soluble solids, amino acids, and ethanol concentrations were similar in four tea wines. Compared to the other three tea wines, GTW had the highest consumption of TSSC and amino acids, and produced the highest alcohol concentration. Therefore, green tea was found more conducive to yeast fermentation and alcohol production compared to the other three tea leaves. As a non-oxidized tea, green tea retained more nutrients during tea processing, such as amino acids and vitamins (23), which might promote the degree of tea wine fermentation. Similar results (14, 24) were found in other fermented products with different tea leaves as raw materials.

### 3.2. The changes of pH and organic acids concentrations during fermentation

**Figure 2:**
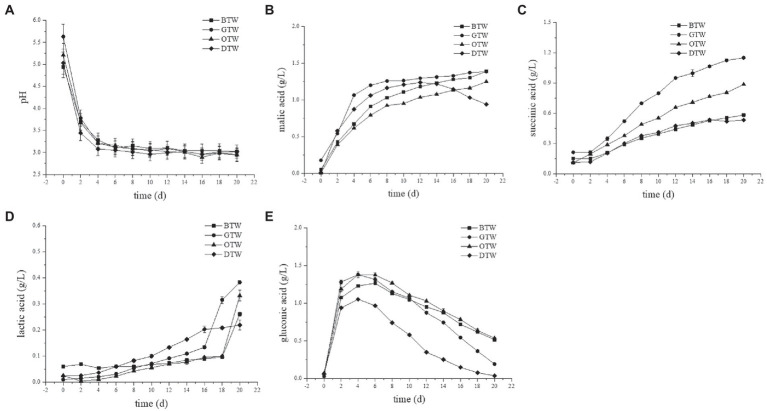
The changes of pH **(A)**, malic **(B)**, succinic **(C)**, lactic **(D)**, and gluconic **(E)** acid concentrations during the fermentation of tea wines.

The changes of pH showed similar trends in four tea wines (Figure 2A). In the first 4 days, the pH of tea wines dropped rapidly to 3.08–3.28, and then slowly decreased and maintained at about 3. The main reason for the decrease of pH was probably due to the accumulation of organic acids during tea wine fermentation (25).

The changes in concentrations of organic acids during the tea wine fermentation were shown in Figures 2B–E. Malic, succinic, lactic, and gluconic acid were the main organic acids found in tea wines, which showed different trends during the fermentation process. The concentrations of malic acid increased rapidly in the first 6 days, and thereafter increased slowly in all tea wines except DTW, which decreased after 12 days. It reached a maximal concentration of 1.39 g/L in BTW and GTW at day 20, which was 1.11-fold and 1.49-fold than that of OTW and DTW, respectively. The concentrations of succinic acid in all types of tea wines increased with prolonged fermentation time, and reached to the maximum of 1.15 g/L in GTW at day 20, which was 1.97-fold, 1.3-fold, and 2.16-fold than that of BTW, OTW, and DTW, respectively. The concentrations of lactic acid increased slowly in the first and middle period, and increased rapidly in the last few days. The concentration of lactic acid was relatively low, and reached to the maximum of 0.38 g/L in GTW at day 20. The concentrations of gluconic acid increased rapidly in the first 4 days, and then gradually decreased in a low range (0.19–0.54 g/L).

After fermentation, GTW showed the highest concentration of malic, succinic, and lactic acid among all tea wines, while BTW had the highest concentration of gluconic acid. The highest concentration of organic acids accumulated in GTW may because its raw materials can promote yeast fermentation. The sensory quality of each organic acid is different (26). Malic acid shows smooth tartness and gluconic acid has mild, soft, and refreshing taste. However, lactic acid presents acrid taste, and succinic acid has slightly bitterness in aqueous solutions. Therefore, the difference of organic acids contents might affect the sensory properties of each tea wines. Moreover, some studies found that the formation of organic acids can improve the antibacterial activity of tea wine (27).

### 3.3. Analysis of volatile components in tea wines

**Figure 3:**
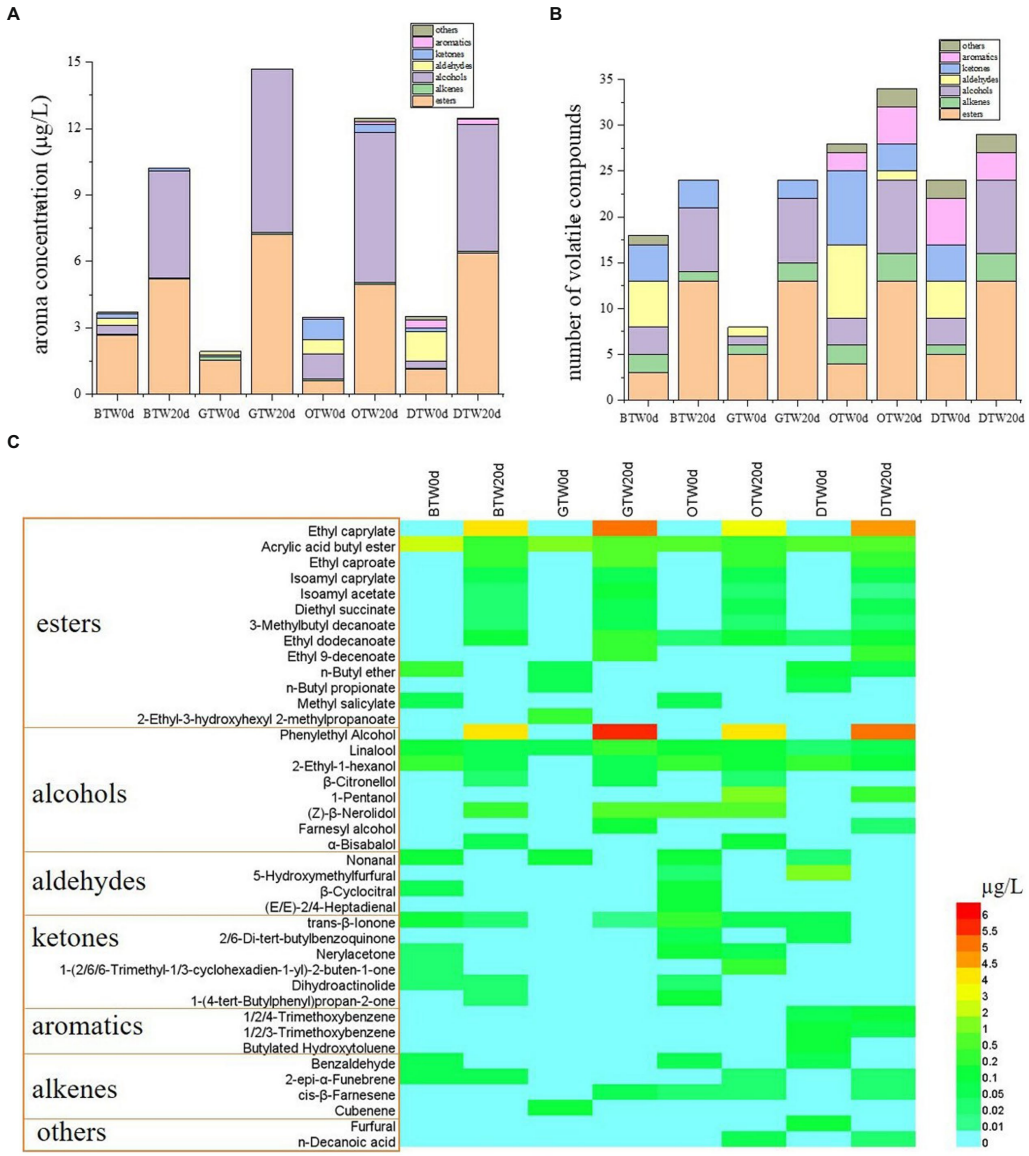
The aroma concentration **(A)**, the number of volatile compounds **(B)**, and the heatmap **(C)** of tea wines.

The volatile compounds of all tea wines were detected by HS-SPME-GC-MS. There were 80 compounds were putatively identified, which was listed in “Supplementary material.” As shown in Figures 3A,B, there were six groups of volatiles identified, including esters, alkenes, alcohols, aldehydes, ketones, and aromatics. After 20 days of fermentation, the aromatics concentrations of four tea wines significantly increased to 10.2–14.7 μg/L from a low level (1.93–3.70 μg/L), especially in esters and alcohols. However, the concentrations of aldehydes significantly decreased after fermentation. Moreover, the number of volatile compounds in all tea wines increased significantly compared to the related tea broth before fermentation. The aromatics concentration of GTW was the highest among four tea wines, while OTW showed the highest number of volatile compounds-34, which was 1.42-fold, 1.42-fold, and 1.17-fold than that of BTW, GTW, and DTW, respectively.

The heatmap of the top 40 compounds ranking their relative concentrations was constructed to visualize the critical metabolites changes of all tea wines (Figure 3C). There were plenty of new esters produced in all tea wines after fermentation, including ethyl caprylate, ethyl caproate, isoamyl caprylate, isoamyl acetate, diethyl succinate, and 3-methylbutyl decanoate. Among them, ethyl ester octanoic acid presented the highest concentration that ranged from 3.82–5.22 μg/L. In terms of alcohols, phenylethyl alcohol was newly produced in all tea wines after fermentation, of which the concentration ranged from 4.22–5.98 μg/L. Moreover, most aldehydes could not be detected after fermentation, which remained a relatively low level of initial concentration.

In general, the number and concentration of esters and alcohols increased significantly after fermentation, while most aldehydes disappeared in tea wines. Ethyl caprylate and phenylethyl alcohol had the highest concentration among all esters and alcohols produced in all tea wines. As reported (28), ethyl caprylate is found as an important aroma contribution of Baijiu, which is generally regarded as fragrant contributor. Phenylethyl alcohol is an aromatic alcohol with rose-like fragrance, usually formed in yeast fermentation (29). Therefore, the accumulation of ethyl caprylate and phenylethyl alcohol might effectively improve the aroma quality of tea wine.

### 3.4. Analysis of catechins and antioxidant activity

As important active components and antioxidants in tea wine, catechins were determined by HPLC and the results were shown in Table 1. The concentration of total catechins in GTW was 404 mg/L, which was 26.5-fold, 1.8-fold, and 64.7-fold than that of BTW, OTW, and DTW, respectively. The concentrations of epi form of catechins (EC, EGC, ECG, and EGCG) in GTW was the highest among all tea wines, while OTW showed the highest concentrations of non-epi form (GC, C, CG, and GCG) in all samples. Eight catechins could be detected in GTW and OTW, while GC, EGC, and EC were not detected in BTW, and GC, EGC, and EGCG were not detected in DTW. The catechin concentrations in BTW and DTW were much lower than those in GTW and OTW. Because black tea is full-fermented tea and dark tea is post-oxidized tea, the catechins were oxidized or degraded in tea processing (30).

**Table 1:** The concentrations of catechins in tea wine samples (mg/L).

	BTW	GTW	OTW	DTW
GC	N.D.	31.3 ± 0.6^b^	50.2 ± 4.2^a^	N.D.
EGC	N.D.	6.6 ± 0.5^b^	14.4 ± 0.8^a^	N.D.
C	3.4 ± 0^c^	19.6 ± 1.2^a^	6.4 ± 0.5^b^	1.8 ± 0^d^
EC	N.D.	2.8 ± 0.6^b^	6.1 ± 0.1^a^	2.1 ± 0.1^c^
EGCG	3.9 ± 0^c^	160 ± 2^a^	92.6 ± 1.1^b^	N.D.
GCG	1.5 ± 0.2^c^	105 ± 6^a^	34.0 ± 1.4^b^	0.3 ± 0^d^
ECG	5.0 ± 0^c^	68.3 ± 1.8^a^	22.0 ± 0.8^b^	1.8 ± 0.2^d^
CG	1.5 ± 0.1^c^	10.9 ± 0.5^a^	2.0 ± 0.2^b^	0.2 ± 0^d^
Non-epi	3.4 ± 0^d^	60.3 ± 2.9^b^	77.2 ± 5.6^a^	3.9 ± 0.1^c^
epi	11.9 ± 0.3^c^	343 ± 10^a^	151 ± 3.5^b^	2.3 ± 0.2^d^
Total	15.2 ± 0.3^c^	404 ± 13^a^	228 ± 9.1^b^	6.2 ± 0.4^d^

Data are means (±SD) of three replicates. Different letters (a, b, c, d) in the same row indicate significant differences between mean values (p < 0.05). N.D., not detected.

**Figure 4:**
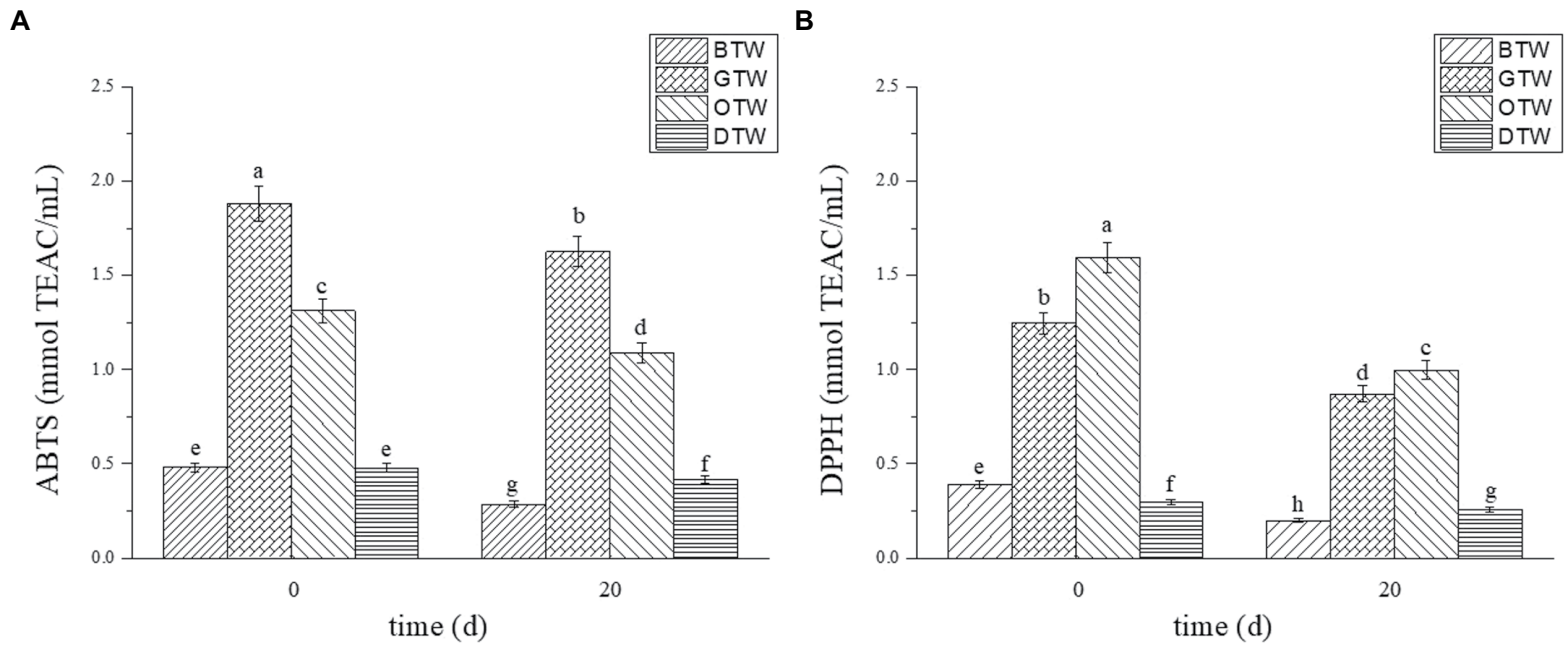
The antioxidant evaluation assays of ABTS **(A)** and DPPH **(B)** scavenging abilities on tea wines. Different letters (a, b, c, d, e, f, g, h) in the same column indicate significant differences between the mean values (*p* < 0.05).

The antioxidant ability of tea wines was analyzed by two different antioxidant evaluation assays of ABTS and DPPH scavenging abilities, and the results were shown in Figure 4. The antioxidant activities determined by ABTS and DPPH assay were decreased significantly in all four types of tea wines at day 20 compared to that of day 0. The reason for the decline of antioxidant activities may be that some antioxidant substances, such as tea polyphenols, were degraded in tea wine fermentation. Similar results were reported in previous research (31, 32).

The effects of tea varieties on antioxidant activities of tea wines were compared. GTW presented the highest ABTS value (1.63 mmol TEAC/ml), which was 5.69-fold, 1.49-fold, and 3.90-fold than that of BTW, OTW, and DTW, respectively. However, OTW showed the highest DPPH value (1.00 mmol TEAC/ml), which was 5.02-fold, 1.15-fold, and 3.89-fold than that of BTW, GTW, and DTW, respectively. The antioxidant abilities of GTW and OTW were much higher than those of BTW and DTW. It was probably due to the higher concentrations of catechins in GTW and OTW, which were mainly responsible for antioxidant activities. Many researchers (33, 34) have reported the similar results.

### 3.5. Evaluation of sensory properties

**Figure 5:**
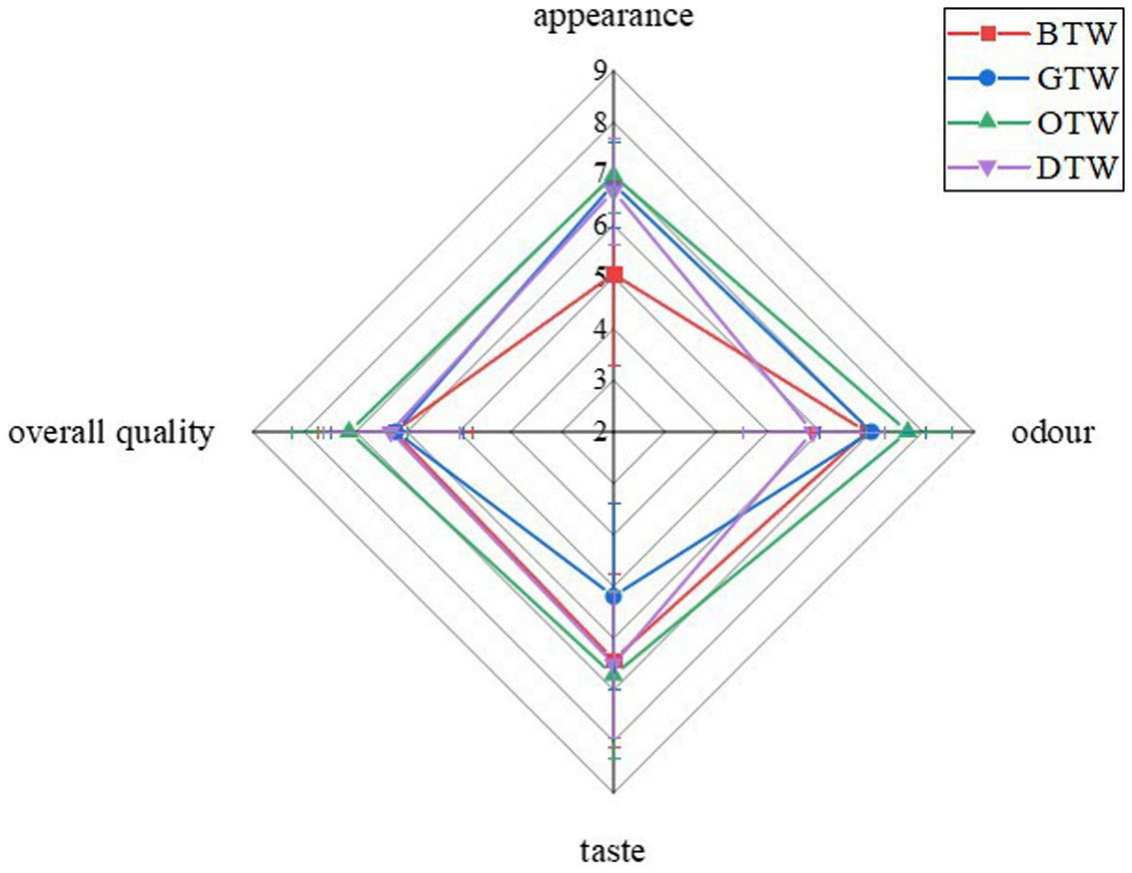
The sensory property evaluation on tea wines.

As shown in Figure 5, the sensory attribute of taste, odor, appearance, and overall acceptability for four types of tea wines were evaluated. The score for taste of OTW was 6.72, which was 4.7, 29.6, and 3.4% higher than that of BTW, GTW, and DTW, respectively. GTW showed the lowest score for taste, probably because the highest concentration of organic acids accumulated, which caused the tea wine to be too sour and taste incongruous. The score for odor of OTW was 7.71, which was 11.6, 10.2, and 31.4% higher than that of BTW, GTW, and DTW, respectively. This may because OTW has the highest number of volatile compounds and the second highest aromatics concentrations. The score for appearance of OTW was 6.97, which was 37.8, 2.5, and 4.5% higher than that of BTW, GTW, and DTW, respectively.

As OTW showed the highest score in taste, odor, and appearance, the score for overall acceptability of OTW was also the highest in all samples, which was 7.13. Therefore, oolong tea is the most suitable for the production of tea wine in terms of flavor. As reported (35, 36), oolong tea was found to be more suitable for many fermented foods than other tea leaves due to its flavor improvement.

## 4. Conclusion

In this research, the effects of tea leaves categories on the physicochemical, antioxidant, and sensorial profile of tea wines were investigated. The dynamic changes of total soluble solids, amino acids and ethanol concentrations, and pH were similar in four tea wines. The GTW showed the highest consumption of total soluble solids and amino acids, and produced the highest concentrations of alcohol, malic, succinic, and lactic acid among all tea wines, which indicated that green tea may promote the degree of tea wine fermentation. The number and concentration of esters and alcohols increased significantly after fermentation, while most aldehydes disappeared in tea wines. Ethyl caprylate and phenylethyl alcohol had the highest concentration among all esters and alcohols produced in all tea wines, which might effectively improve the aroma quality of tea wine. GTW presented the highest volatile concentration, while oolong tea wine (OTW) showed the highest number of volatile compounds. GTW had the highest total catechins concentration of 404 mg/L and the highest ABTS value (1.63 mmol TEAC/ml), while OTW showed the highest DPPH value (1.00 mmol TEAC/ml). Moreover, OTW showed the highest score in taste, odor, appearance, and overall acceptability, which indicated that oolong tea is the most suitable for the production of tea wine in terms of flavor. Therefore, the types of tea leaves used in the tea wine production interfere in its bioactive composition, sensorial, and antioxidant properties. This study provides guidance for the selection of tea leaves as the raw materials in the tea wine production.

## Data availability statement

The raw data supporting the conclusions of this article will be made available by the authors, without undue reservation.

## Author contributions

CZ: conceptualization, formal analysis, investigation, data curation, writing – original draft, and funding acquisition. D-QC and H-FH: formal analysis, investigation, and data curation. Y-BH and Z-HF: investigation and data curation. J-XC and FW: investigation. Y-QX and J-FY: writing – review and editing, supervision, funding acquisition, and project administration. All authors contributed to the article and approved the submitted version.

## Funding

This research was supported by the National Natural Science Foundation of China (32002094), the Key Research and Development Program of Zhejiang (2022C02033), the China Agriculture Research System of MOF and MARA (CARS-19) and the Innovation Project for Chinese Academy of Agricultural Sciences (CAAS-ASTIP-TRI).

## Conflict of interest

The authors declare that the research was conducted in the absence of any commercial or financial relationships that could be construed as a potential conflict of interest.

## Publisher’s note

All claims expressed in this article are solely those of the authors and do not necessarily represent those of their affiliated organizations, or those of the publisher, the editors and the reviewers. Any product that may be evaluated in this article, or claim that may be made by its manufacturer, is not guaranteed or endorsed by the publisher.

## Supplementary material

The Supplementary material for this article can be found online at: https://www.frontiersin.org/articles/10.3389/fnut.2023.1110803/full#supplementary-material
