# Supplementary material for: Impact of tea leaves categories on physicochemical, antioxidant, and sensorial profiles of tea wine
Source: Front Nutr. 2023 Feb 7;10:1110803. doi: 10.3389/fnut.2023.1110803 (PMC9941558; doi:10.3389/fnut.2023.1110803)
Supplement: Supplementary file 1 [file Table_1.DOCX]

Supplementary Material

Impact of tea leaves categories on physicochemical, antioxidant, and sensorial profiles of tea wine

Chun Zou, De-Quan Chen, Hua-Feng He, Yi-Bin Huang, Zhi-Hui Feng, Jian-Xin Chen, Fang Wang, Yong-Quan Xu*, Jun-Feng Yin**

*** Correspondence:** Yong-Quan Xu: [yqx33@126.com](mailto:yqx33@126.com); Jun-Feng Yin: yinjf@tricaas.com.

# Supplementary Data

| No. | Name | CAS | RI | | relative concentration (µg/L) to internal standard | | | | | | | |
| --- | --- | --- | --- | --- | --- | --- | --- | --- | --- | --- | --- | --- |
|  |  |  | Exp. | Ref. | BTW0d | BTW20d | GTW0d | GTW20d | OTW0d | OTW20d | DTW0d | DTW20d |
| 1 | 1-Pentanol | 71-41-0 | 733 | 730 |  |  |  |  |  | 1.07 |  | 0.35 |
| 2 | Furfural | 98-01-1 | 842 | 840 |  |  |  |  | 0.01 |  | 0.12 |  |
| 3 | Isoamyl acetate | 123-92-2 | 877 | 877 |  | 0.03 |  | 0.14 |  | 0.04 |  | 0.01 |
| 4 | n-Butyl ether | 142-96-1 | 883 | 888 | 0.23 |  | 0.07 |  |  |  | 0.13 | 0.05 |
| 5 | Ethanol, 2-butoxy- | 111-76-2 | 886 | 885 | 0.04 |  |  |  |  |  |  |  |
| 6 | Acrylic acid butyl ester | 141-32-2 | 896 | 892 | 2.29 | 0.44 | 1.07 | 0.56 | 0.50 | 0.34 | 0.87 | 0.65 |
| 7 | n-Butyl propionate | 590-01-2 | 908 | 908 |  |  | 0.06 |  |  |  | 0.05 |  |
| 8 | Benzaldehyde | 100-52-7 | 971 | 970 | 0.09 |  |  |  | 0.06 |  | 0.05 |  |
| 9 | Butanoic acid, butyl ester | 109-21-7 | 997 | 996 |  |  |  |  | 0.04 |  | 0.04 |  |
| 10 | Ethyl caproate | 123-66-0 | 998 | 999 |  | 0.32 |  | 0.52 |  | 0.27 |  | 0.28 |
| 11 | (E,E)-2,4-Heptadienal | 4313-03-5 | 1017 | 1017 |  |  |  |  | 0.16 |  |  |  |
| 12 | 2-Ethyl-1-hexanol | 104-76-7 | 1033 | 1031 | 0.24 | 0.05 |  | 0.07 | 0.26 | 0.10 | 0.25 | 0.15 |
| 13 | Benzeneacetaldehyde | 122-78-1 | 1052 | 1053 |  |  |  |  | 0.05 |  |  |  |
| 14 | 3,5-Octadien-2-one | 38284-27-4 | 1075 | 1074 |  |  |  |  | 0.06 |  |  |  |
| 15 | 6-Methyl-2-pyrazinylmethanol | 77164-93-3 | 1086 | 1084 |  |  |  |  |  |  | 0.03 |  |
| 16 | 2,5-Furandicarboxaldehyde | 823-82-5 | 1089 | 1084 |  |  |  |  |  |  | 0.02 |  |
| 17 | 2-Nonanone | 821-55-6 | 1092 | 1093 |  |  |  | 0.01 |  |  |  |  |
| 18 | Heptanoic acid, ethyl ester | 106-30-9 | 1097 | 1097 |  | 0.03 |  | 0.02 |  | 0.02 |  |  |
| 19 | Linalool | 78-70-6 | 1101 | 1101 | 0.13 | 0.06 | 0.08 | 0.21 | 0.13 | 0.10 | 0.04 | 0.05 |
| 20 | Nonanal | 124-19-6 | 1106 | 1107 | 0.17 |  | 0.16 |  | 0.15 |  | 0.03 |  |
| 21 | Phenylethyl Alcohol | 60-12-8 | 1118 | 1117 |  | 4.22 |  | 5.98 |  | 4.35 |  | 5.10 |
| 22 | Benzyl nitrile | 140-29-4 | 1145 | 1143 |  |  |  |  |  | 0.02 |  |  |
| 23 | Pyranone | 28564-83-2 | 1149 | 1154 |  |  |  |  |  |  | 0.01 |  |
| 24 | Acetic acid, 2-ethylhexyl ester | 103-09-3 | 1149 | 1149 |  |  | 0.04 |  |  |  |  |  |
| 25 | Octanoic Acid | 124-07-2 | 1177 | 1177 |  |  |  |  |  | 0.09 |  | 0.01 |
| 26 | Diethyl succinate | 123-25-1 | 1179 | 1179 |  | 0.02 |  | 0.08 |  | 0.08 |  | 0.08 |
| 27 | Naphthalene | 91-20-3 | 1193 | 1193 |  |  |  |  | 0.02 | 0.03 | 0.03 |  |
| 28 | Ethyl caprylate | 106-32-1 | 1196 | 1195 |  | 4.06 |  | 5.22 |  | 3.82 |  | 4.60 |
| 29 | Methyl salicylate | 119-36-8 | 1200 | 1199 | 0.07 |  |  |  | 0.05 |  |  |  |
| 30 | β-Safranal | 116-26-7 | 1204 | 1205 | 0.03 |  |  |  | 0.02 |  |  |  |
| 31 | Decanal | 112-31-2 | 1208 | 1208 | 0.03 |  |  |  | 0.03 |  |  |  |
| 32 | β-Cyclocitral | 432-25-7 | 1226 | 1226 | 0.06 |  |  |  | 0.11 |  |  |  |
| 33 | β-Citronellol | 106-22-9 | 1229 | 1220 |  | 0.04 |  | 0.07 |  | 0.04 |  |  |
| 34 | Benzothiazole | 95-16-9 | 1237 | 1236 |  |  |  |  |  | 0.04 |  |  |
| 35 | 5-Hydroxymethylfurfural | 67-47-0 | 1243 | 1241 |  |  |  |  | 0.04 |  | 1.26 |  |
| 36 | Benzeneacetic acid, ethyl ester | 101-97-3 | 1247 | 1247 |  | 0.02 |  | 0.04 |  |  |  | 0.01 |
| 37 | Isopentyl hexanoate | 2198-61-0 | 1252 | 1252 |  |  |  | 0.02 |  | 0.03 |  |  |
| 38 | Geraniol | 106-24-1 | 1254 | 1254 |  |  |  | 0.05 |  |  |  |  |
| 39 | β-Cyclohomocitral | 472-66-2 | 1263 | 1261 |  |  |  |  | 0.08 |  |  |  |
| 40 | 1,2,3-Trimethoxybenzene | 634-36-6 | 1309 | 1315 |  |  |  |  |  |  | 0.10 | 0.06 |
| 41 | Naphthalene, 2-methyl- | 91-57-6 | 1324 | 1318 |  |  |  |  |  |  | 0.03 |  |
| 42 | n-Caprylic acid isobutyl ester | 5461-06-3 | 1348 | 1348 |  | 0.01 |  | 0.01 |  | 0.01 |  | 0.02 |
| 43 | Dehydro-ar-ionene | 30364-38-6 | 1365 | 1364 |  |  |  |  | 0.05 |  |  |  |
| 44 | 2(3H)-Furanone, dihydro-5-pentyl- | 104-61-0 | 1367 | 1366 |  | 0.02 |  |  |  |  |  |  |
| 45 | 1,2,4-Trimethoxybenzene | 135-77-3 | 1372 | 1374 |  |  |  |  |  |  | 0.05 | 0.15 |
| 46 | 2-Ethyl-3-hydroxyhexyl 2-methylpropanoate | 74367-31-0 | 1378 | 1375 |  |  | 0.31 |  |  |  |  |  |
| 47 | n-Decanoic acid | 334-48-5 | 1380 | 1380 |  |  |  |  |  | 0.08 |  | 0.03 |
| 48 | 1-(2,6,6-Trimethyl-1,3-cyclohexadien-1-yl)-2-buten-1-one | 23696-85-7 | 1385 | 1362 | 0.05 |  |  |  |  | 0.25 |  |  |
| 49 | Ethyl 9-decenoate | 067233-91-4 | 1386 | 1386 |  |  |  | 0.24 |  |  |  | 0.43 |
| 50 | (E)-α-Ionone | 127-41-3 | 1429 | 1429 | 0.02 |  |  |  |  |  | 0.04 |  |
| 51 | 2-epi-α-Funebrene | 469-61-4 | 1431 | 1431 | 0.05 | 0.05 |  |  |  | 0.04 |  | 0.03 |
| 52 | 1-(4-tert-Butylphenyl)propan-2-one | 81561-77-5 | 1435 | 1455 |  | 0.04 |  |  | 0.16 |  |  |  |
| 53 | Isoamyl caprylate | 2035-99-6 | 1447 | 1446 |  | 0.05 |  | 0.06 |  | 0.06 |  | 0.05 |
| 54 | Nerylacetone | 3879-26-3 | 1451 | 1455 | 0.04 |  |  |  | 0.14 | 0.05 |  |  |
| 55 | cis-β-Farnesene | 28973-97-9 | 1455 | 1455 |  |  |  | 0.05 | 0.03 | 0.04 |  | 0.04 |
| 56 | 2,6-Di-tert-butylbenzoquinone | 719-22-2 | 1469 | 1468 |  |  |  |  | 0.08 |  | 0.05 |  |
| 57 | Dehydro-β-ionone | 1203-08-3 | 1483 | 1485 |  |  |  |  | 0.01 |  |  |  |
| 58 | trans-β-Ionone | 14901-07-6 | 1486 | 1486 | 0.10 | 0.03 |  | 0.01 | 0.42 | 0.06 | 0.05 |  |
| 59 | β-Ionone epoxide | 23267-57-4 | 1489 | 1488 |  |  |  |  | 0.04 |  |  |  |
| 60 | Undecanoic acid, ethyl ester | 627-90-7 | 1493 | 1491 |  | 0.03 |  |  |  | 0.03 |  | 0.01 |
| 61 | .beta.-D-Glucopyranose, 1,6-anhydro- | 498-07-7 | 1499 | 1491 |  |  |  |  |  |  | 0.06 |  |
| 62 | (E,E)-α-Fernesene | 502-61-4 | 1505 | 1505 |  |  |  | 0.02 |  |  |  | 0.01 |
| 63 | Butylated Hydroxytoluene | 128-37-0 | 1507 | 1505 |  |  |  |  |  |  | 0.15 |  |
| 64 | 2,4-Di-tert-butylphenol | 96-76-4 | 1508 | 1509 |  |  |  |  |  |  |  | 0.03 |
| 65 | β-Bisabolene | 495-61-4 | 1513 | 1513 |  |  |  |  |  | 0.02 |  |  |
| 66 | Cubenene | 483-76-1 | 1527 | 1528 |  |  | 0.15 |  |  |  |  |  |
| 67 | Lilial | 80-54-6 | 1535 | 1535 |  |  |  |  |  | 0.01 |  |  |
| 68 | Dihydroactinolide | 15356-74-8 | 1546 | 1538 | 0.04 | 0.02 |  |  | 0.04 |  |  |  |
| 69 | Dihydroactinidiolide | 17092-92-1 | 1546 | 1546 |  |  |  |  |  |  | 0.03 |  |
| 70 | (Z)-β-Nerolidol | 142-50-7 | 1566 | 1565 |  | 0.38 |  | 0.86 | 0.72 | 0.95 |  |  |
| 71 | Z,E-Nerolidol | 7212-44-4 | 1571 | 1571 |  | 0.04 |  |  |  |  |  |  |
| 72 | trans-β-Nerolidol | 40716-66-3 | 1572 | 1572 |  |  |  |  |  | 0.03 |  | 0.02 |
| 73 | Ethyl dodecanoate | 106-33-2 | 1592 | 1592 |  | 0.12 |  | 0.24 | 0.03 | 0.16 | 0.03 | 0.15 |
| 74 | Cedrol | 77-53-2 | 1632 | 1625 | 0.03 |  |  |  |  |  |  | 0.01 |
| 75 | Benzene, (1-butylheptyl)- | 4537-15-9 | 1636 | 1633 |  |  |  |  |  | 0.01 |  |  |
| 76 | 3-Methylbutyl decanoate | 2306-91-4 | 1647 | 1647 |  | 0.02 |  | 0.06 |  | 0.03 |  | 0.03 |
| 77 | α-Bisabalol | 515-69-5 | 1698 | 1697 |  | 0.06 |  |  |  | 0.10 |  |  |
| 78 | Farnesyl alcohol | 4602-84-0 | 1725 | 1724 |  |  |  | 0.15 |  |  |  | 0.03 |
| 79 | (Z,E)-Farnesol | 3790-71-4 | 1730 | 1732 |  |  |  |  |  |  |  | 0.02 |
| 80 | Diisobutyl phthalate | 84-69-5 | 1865 | 1863 |  |  |  |  |  | 0.07 |  |  |

Note: RI means retention index; Exp. means experiment; Ref. means reference.
